# Epidemiology of tuberculosis in children in Kampala district, Uganda, 2009–2010; a retrospective cross-sectional study

**DOI:** 10.1186/s12889-015-2312-2

**Published:** 2015-09-25

**Authors:** Eric Wobudeya, Deus Lukoye, Irene R. Lubega, Frank Mugabe, Moorine Sekadde, Philippa Musoke

**Affiliations:** Directorate of Paediatrics & Child health, Mulago National Referral Hospital, P. O. Box 7051, Kampala, Uganda; MU-JHU Research Collaboration, P. O. Box 23491, Kampala, Uganda; TRACK TB Project, Management Sciences for Health, P. O. Box 71419, Kampala, Uganda; Uganda National TB & Leprosy Programme, P. O. Box 7272, Kampala, Uganda; Department of Paediatrics & Child Health, Makerere University College of Health Sciences, P. O. Box 7062, Kampala, Uganda

## Abstract

**Background:**

The global tuberculosis (TB) estimate in 2011 was 500,000 cases among children under 15 years representing 5.7 % of all cases and 64, 000 deaths among HIV negative children representing 6.5 % of the total deaths. In Uganda, the child TB cases reported in 2012 made up less than 3 % of the total cases while recent modelling estimates it at 15–20 % of adult cases. Mapping of these cases in Kampala district most especially for the children under five years would reflect recent transmission in the various communities in the district. We therefore conducted a retrospective study of reported child TB cases in Kampala district Uganda for 2009–2010 to provide an estimate of child TB incidence and map the cases.

**Methods:**

This was a retrospective cross-sectional study on data collected from the health unit TB registers in the five divisions of Kampala district, Uganda. The data was a starting point in preparation for a TB Vaccine study in children. The extracted data spanned a period from 1st January 2009 to 31st December 2010. The projected population of children below 15 years was 637,922 in 2009 and 744,750 in 2010 for Kampala district. We based our projections on the National Bureau of Statistics most recent census report of 2002 before the study duration while assuming a population growth rate of 3.7 % each year. We captured the data into EPI DATA 3.1 and analysed it using STATA version 12.

**Results:**

We accessed 15,499 records and analysed 1167 records that were of children below 15 years old. The child TB cases represented 7.5 % (7.3 in 2009 & 7.6 % in 2010) of all the registered cases in Kampala district. The females were 47 % and the median age was 4 years (IQR 1, 10). The percent of children less than 5 years old was 54 %. The percent of pulmonary TB cases was 89 % (1041/1167) with 15 % smear positive. The proportion of extra-pulmonary TB cases was 11 % (126/1167). Among those that tested for HIV, 60 % (359/620) had test results available with an HIV co-infection rate of 47 % (168/359). Antiretroviral treatment uptake was 24 % among the co-infected. The incidence of child TB in Kampala was 56 (95 % CI 50–62) per 100,000 in 2009 and 44 (95 % CI 40–49) per 100,000 in 2010. Most of the TB cases (60 % (410/685)) in Kampala live in slum areas.

**Conclusion:**

There was a higher child TB incidence of 56 per 100,000 in 2009 compared with 44 per 100,000 in 2010. The percentage of child TB cases was much higher at 7.5 % of all the reported TB cases than the WHO reported national average. For the review period, the TB cases clustered in particular slums in Kampala district.

## Background

In 2011, the global TB estimate was 8.7 million incident cases with the African region accounting for 26 % and 1.4 million deaths including 430, 000 deaths among HIV-positive people [1]. The 2011 estimates represented for the first time the expanded inclusion of data in children beyond smear positive cases. In the same report, there were an estimated 500, 000 cases among children under 15 years old representing 5.7 % of all cases and 64, 000 deaths in children 6.5 % of the global deaths [1]. Until the global tuberculosis report of 2012, reported PTB cases were the smear positives and therefore included few children. Children mainly have paucibacillary (low TB germ load) disease so are usually smear negative. Consequent to the paucibacillary nature of TB in children, the true burden of TB disease in children remains uncertain.

A recent mathematical modelling estimated about 650,000 incident TB cases in children under 15 years old in 2010 and about 7.5 Million TB infections [2]. There were however no global estimates for the 2010 incident TB cases in children for comparison. The model further estimated that 4–20 % of global TB cases occurs in children while for Uganda it estimated that 15–20 % the cases should be among children [2]. The challenge of childhood TB diagnosis coupled with misdiagnosis of extra-pulmonary TB contributes to uncertain estimates.

Although TB and HIV epidemics link, the drop of 50 % in HIV infection between 2001 and 2012 [3] does not compare with the 2 % decline in TB incidence between 1990 and 2012 [4]. Most of the TB decline is in South-East Asia specifically China. Uganda has registered a remarkable decline in the prevalence of TB and is on track to meet the Millennium Development Goal (MDG) target [5]. We do not know if the decline is in all communities or a few selected population areas. There is a high risk of TB disease in exposed children most especially those with HIV infection and severe malnutrition [6, 7]. The interplay between TB, poverty and overcrowding [8] leads to varying trends in different populations.

In Uganda, the TB cases reported in children in 2012 made up less than 3 % of the total reported TB cases [4]. There is a high likelihood of TB under-reporting in children because most are diagnosed on clinical basis as opposed to bacteriologically confirmed. We also expect the numbers in children to be a reflection of adult numbers since their TB disease is commonly from adults. Tuberculosis in under fives represents recent transmission reflected in mapping of cases in communities [9]. Until 2012, the World TB reports data for Uganda did not provide information on all forms of childhood TB reported. Age broken down data and TB cases location information is important for targeted interventions and may guide the choice of TB research populations.

The specific objective this cross-sectional retrospective study was to provide an estimate of incidence and distribution of childhood TB in Kampala district, Uganda.

## Methods

### Study design

This was retrospective study on data collected from health unit TB registers as starting data in preparation for a TB vaccine study in children.

### Setting

We collected data from the five administrative divisions of Kampala district. Kampala city, the capital city of Uganda is located in Kampala district. It has an estimated population of 1.5 million by night and much higher by day due to large numbers that come to work. Kampala district is surrounded by Wakiso district from where many people travel to work in the capital city. The district has 62 informal settlements referred to as slums. The district had a projected population of children below 15 years of 637,922 in 2009 and 744,750 in 2010. The projection was from the National Bureau of Statistics 2002 population census report (which was the most recent before the study period) assuming a constant annual population growth rate of 3.7 % [10]. During the study period, TB surveillance in most of the health unit was mainly passive. In the passive surveillance patients present to the health unit for TB related symptoms. In active TB surveillance patients presenting to the health unit for any reason are screened for TB. Active TB surveillance or screening was limited to HIV clinics. There are 52 TB diagnostic and treatment units (DTU) in the Kampala district. Each of the DTUs registers TB cases in the Unit TB registers.

### Data source

We reviewed all extracted routine NTLP data from 1st January 2009 to 31st December 2010 from the health unit TB registers in Kampala district, Uganda. Following the clinician’s diagnosis, unit TB focal person or TB staff record patient data in paper based unit TB registers. We used the national guideline definitions as adapted from WHO TB guidance of 2006 for case classification during the review period [11]. A case of TB was one with bacteriological confirmation (sputum smear positive or culture) or where a clinician decided to treat for TB. Each TB case is reported as pulmonary TB (smear positive or smear negative or smear not done) or extra pulmonary TB. Since not all children had sputum collected, we included PTB smear not done as a category for intervention purposes. All the TB cases are offered an opportunity to test for HIV as part of the routine tests.

### Data management and statistical analysis

We captured the data in EPI DATA 3.1 and analysed using the STATA version 12. We described continuous data using medians with inter-quartile ranges while categorical data as proportions. We present the data in tables and graphs. We only included cases of children residing in Kampala district at registration time to calculate incidence rates for Kampala district.

### Ethical considerations

The study received ethical approval from Mulago Hospital Research and Ethics committee and consent obtained from the NTLP of the Ministry of Health. We only extracted non-identifying data.

## Results

We accessed 15,499 patient TB records and extracted 1167 records of children less than 15 years for analysis.

### Descriptive data

Children accounted for 7.5 % (7.3 in 2009 & 7.6 % in 2010) of all the reported TB cases in Kampala. The median age was 4 years (IQR 1, 10) and the majority (54 %) were under 5 years old with 47 % (548/1167) being females. See Table 1 for other demographic characteristics. Most of the children were 0–4 years making up 54 %, those 5–9 years made up 21 % and the 10–14 years were 25 %. The TB cases residing in Kampala were 59 % while those residing outside Kampala district were 41 % (Wakiso −20 %, elsewhere− 21 %).

**Table 1:** Demographic characteristics of the children with TB notified in Kampala district, Uganda 2009–2010 (*N* = 1167)

Characteristic		Frequency of TB cases	Percentage
Age group in years	0–4	629	54 %
	5–9	246	21 %
	10–14	292	25 %
District	Kampala	685	59 %
	Wakiso	238	20 %
	Others	244	21 %
Division	Kawempe	172	25 %
	Makindye	139	20 %
	Central	38	5.6 %
	Nakawa	120	17 %
	Rubaga	216	31 %
Sex	Female	548	47 %
	Male	619	53 %
TB type	PTB	1041	89 %
	EPTB	126	11 %
Sputum collected	Yes	456	39 %
	No	711	61 %

Seventy five percent (874/1167) had HIV test counselling, 71 % (620/874) were tested and results were available for 60 % (359/620). More children under five had unknown HIV status than other age groups (57 % vs 48 %). Table 2 shows some other characteristics of children with known and unknown HIV status. Of those children with available results, 47 % (168/359) were HIV-positive. Twenty four percent (40/168) of the children with HIV co-infection were on antiretroviral therapy (ART) while 84 % (141/168) were on cotrimoxazole prophylaxis therapy (CPT).

**Table 2:** Characteristics of children with known and unknown HIV status notified as TB in Kampala district, Uganda 2009–2010 (*N* = 1167)

Characteristic		HIV test result		
		Known *n* (%)	Unknown *n* (%)	*P* value
Age group in years	0–4	172 (48)	457 (57)	0.023
	5–9	84 (23)	162 (20)	
	10–14	103 (29)	189 (23)	
District	Kampala	210 (59)	475 (59))	0.049
	Wakiso	86 (24)	152 (19)	
	Others	63 (18)	181 (22)	
Division of Kampala district	Central	10 (5)	28 (6)	0.044
	Kawempe	43 (20)	129 (27)	
	Rubaga	74 (35)	142 (30)	
	Makindye	36 (17)	103 (22)	
	Nakawa	47 (22)	73 (15)	
Sex	Female	190 (53)	358 (44)	0.006
	Male	169 (47)	450 (56)	
TB type	PTB	307 (86)	733 (91)	0.011
	EPTB	51 (14)	75 (9)	
Sputum collected	Yes	207 (58)	249 (31)	<0.001
	No	151 (42)	560 (69)	

There were 89 % (1041/1167) PTB cases and 10 % (126/1167) EPTB cases. Among the children with pulmonary TB (PTB), 30 % (308/1041) had HIV test results of which 47 % (144/308) were positive. Among extra-pulmonary TB (EPTB) cases, 40 % (51/126) had HIV test results of which 47 % (24/51) were positive. The HIV positivity rate by age groups was; 47 % in 0–4, 59 % in 5–9 and 26 % in 10–14 years. The differences were statistically significant (see Table 3).

**Table 3:** General characteristics of HIV positive and negative children with TB notified in Kampala district, Uganda, 2009–2010 (*N* = 359)

Characteristic		HIV test result		
		Positive *n *(%)	Negative *n *(%)	*P* value
Age group in years	0–4	81 (48)	91 (48)	<0.002
	5–9	51 (30)	33 (17)	
	10–14	36 (21)	67 (35)	
District	Kampala	98 (58.33	112 (58.64	0.989
	Wakiso	40 (23.81	46 (24.08	
	Others	30 (17.86	33 (17.28	
Division	Kawempe	15 (15.31	28 (25.00	0.158
	Makindye	19 (19.39	17 (15.18	
	Central	4 (4.08	6 (5.36	
	Nakawa			
	Rubaga	32 (32.65	42 (37.50	
Sex	Female	88 (52.38	102 (53.40	0.846
	Male	80 (47.62	89 (46.60	
TB type	PTB	144 (85.7	163 (85.8)	0.983
	EPTB	24 (14.29)	27 (14.21)	
Sputum collected	Yes	103 (61.3)	104 (54.7	0.208
	No	65 (38.69)	86 (45.26 )	

**Fig. 1:**
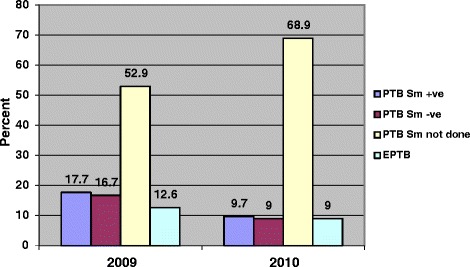
Distribution of TB types in children in Kampala district, Uganda 2009, 2010 (*N* = 1167)

Of the PTB cases, 15 % (160/1041) were sputum acid-fast bacilli smear positive, 16 % (170/1041) were sputum acid-fast bacilli smear negative and 68 % (711/1041) had no smear done (no sputum collected). Among those with smear not done, most (68 %) were below 5 years old. There was a higher number of PTB cases with smear not done in 2010 (69 %) compared with 53 % in 2009 as shown in Fig. 1. The smear positive and smear negative PTB cases as well as EPTB cases decreased between 2009 and 2010 (see Fig. 1). Distribution of the smear positive cases by age group was; 73 % (116/160) among 10–14, 15 % (24/160) among 5–9 and 13 % (20/160) among 0–4 years. Distribution of the EPTB cases by age group was: 36 % (45/126) among 10–14, 37 % (46/126) among 0–4 and 28 % (35/126) among 5–9 years old.

### Main study results

The proportion of TB cases in the 0–4 years age group was 52 in 2009 and 56 % in 2010. In the 10–14 years age group the percent was 27 in 2009 and 24 % in 2010 while in the 5–9 years age group it was 21 in 2009 and 20 % in 2010.

**Fig. 2:**
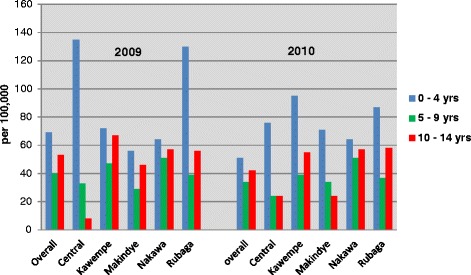
Childhood TB incidence by age group and division in Kampala district, Uganda 2009, 2010

The overall child TB incidence in Kampala was 56 (95 % CI 50–62) per 100,000 in 2009 and 44 (95 % CI 40–49) per 100,000 in 2010. The child TB incidences by age group and division also decreased over the study period as shown in Fig. 2.

**Fig. 3:**
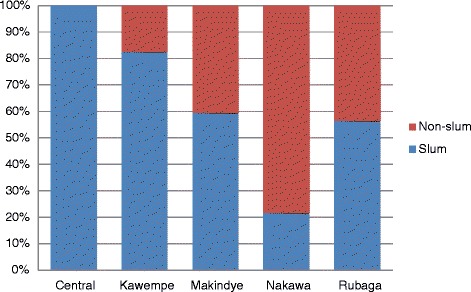
Distribution of residence of TB cases residing in Kampala District, Uganda 2009–2010 (*N* = 685)

Most of the TB cases, 60 % (410/685), in Kampala lived in slum areas. The Fig. 3 shows distribution of TB cases by area and division.

## Discussion

### Main findings

Our findings showed a general decrease in TB incidence with most cases of PTB having no smear done and majority of EPTB cases occurring among adolescents. We found a high rate of smear positive cases of up to 15 %. Our study highlights low HIV test uptake, high absence of HIV test results and large percentage TB cases residing in slum areas in the Kampala district.

### Relation to literature

Our findings showed that many cases registered in Kampala district live outside the Kampala administrative borders. The incident cases registered in Kampala district may therefore not represent the true picture of TB burden within the various communities in Kampala district.

We noted a decrease in TB incidence over the review period similar to that in the World TB report 2011 [12]. The report shows a declining trend in TB incidence in adults. This trend should reflect in children as we know that TB cases in children especially those below 5 years represents recent transmission in the community [13, 14]. Previous work showed that up to 30 % of the children with TB will have an identifiable household source case [15]. Similar to previous reports, our data shows a bimodal distribution with more cases in under five and 10–14 years age groups compared with the 5–9 year olds [16, 17].

Our study found the total number of TB cases had not substantially reduced (the reported TB cases decreased by 8 %) over the review period. The reported decreased incidence may be due to high population growth rate without the proportionate increase in the number of new TB cases. We however report an increased TB incidence in the under fives that we suppose is attributable to improved diagnosis and reporting rather than increasing burden of TB in children. Our report shows that 7.5 % of the TB cases registered in Kampala district were among children. This is much higher than the national average of 1.5 % reported in the world TB report 2013 but less than the estimated expected burden of 15–20 % in the high burden countries [2]. The World TB report 2014 still documented that childhood TB represents 1.5 % of the total cases with no decline in the total number of new cases [18]. We suppose that this discrepancy is due not reporting PTB cases in children with smear negative disease or smear not done. The increased child TB case detection in the under fives is an encouraging finding during this review period. This finding may be a spill over from Tuberculosis control assistance program (TB CAP) activities that included health worker training and provision of tools for TB care [19]. There is evidence that training health workers and provision of the relevant job aids in the diagnosis of children TB increases childhood TB detection rates [20]. Finding more children with no sputum examinations done on the background of several efforts to increase TB detection in children needs innovative ways to cause routine sputum collection from children. The high number of smear not done cases in children is likely due to limited health worker confidence and skills to collect sputum from children. This means most children are diagnosed on clinical basis with no sputum collection. The reality of multi-drug resistant (MDR) TB makes it even more pressing to build capacity for sputum collection from children.

The lowest TB incidence was in the 5–9 year age group, a safer age group as reported in literature [16]. Most of the childhood TB cases (56 %) were in children below 5 years old. This age group has the highest risk of developing TB disease because of low immunity and other reasons such as malnutrition and HIV [8, 21]. This age group is more likely to get exposure for longer periods to infectious adults within their households [22].

We found most TB cases were pulmonary, a finding reported by other studies [23, 24]. A TB report from Zambia showed that about 72 % of childhood TB cases were pulmonary [25]. Even for the children less than five years old we report more PTB cases than EPTB. Literature reports children are more prone to EPTB most especially if HIV positive [26]. However, we report more cases of PTB than EPTB even when the HIV prevalence was high among the tested patients. We found equal proportions of children with HIV among the PTB and EPTB cases. We expect a higher proportion with EPTB in a population of children with high HIV co-infection and low ART uptake at 24 % on the premise that children are prone to disseminated disease if HIV infected. This finding also reported in other settings [23–25] may be related to training of health workers that mainly focuses on PTB. It is possible the EPTB cases are largely missed. Finding about forty per cent EPTB cases among adolescents is unexpected since at this age there is more TB containment outside the lungs. The available data neither included the sites of EPTB nor provided an explanation for this observation. A study of incident TB cases among adolescents in South Africa found only 3 % had EPTB [27]. The total child TB cases reported in the 2010 and 2011 Global TB reports is 1291 for Uganda while we report 1167 child TB cases for Kampala district alone during the same time period. Our report underpins the reality of child TB under-reporting because of the emphasis on reporting smear positive cases only.

We report an overall smear positivity rate of 15 % among those tested and most of these (72 %) were adolescents 10–14 years. We report a high positive smear rate ranging from 13 % in 0–4 years to 15 % among children 10–14 years. The smear positivity rate among adolescents is similar to the adult smear positivity rate of 20 % reported in Uganda in 2007 [28]. This is not surprising since adolescents get adult type pulmonary disease. We report a smear positivity rate of 13 % among those below 5 years. This suggests that routine use of Xpert® MTB/RIF as recommended in this age group would yield many more bacteriologically confirmed cases in the under fives. The Xpert® MTB/RIF is a hands-free sample real-time PCR analysis system, developed under Cepheid (a molecular diagnostics company), that simultaneously detects mycobacterium tuberculosis (MTB) and resistance to rifampicin (RIF). In our previous study in a research setting, Xpert® MTB/RIF identified twice as many cases as microscopy [29].

The low HIV testing and results availability reported in this paper suggests gaps in integration of TB and HIV services. A similar finding was reported in non-integrated TB and HIV services in South Africa where only 26 % of the TB patients knew their HIV status. In the same report, the number tested for HIV was low at diagnosis compared to at 2 or 6 months while on TB treatment [30].

There were many TB cases residing outside Kampala district but registered and treatment in Kampala. This finding may reflect limited confidence and capacity of health workers outside Kampala district to diagnose TB in children. The likely implication is potential increase in TB transmission by caregivers (who are the likely source of TB) during their travel by public transport to access services in Kampala district. This exposure may vary from as short as 10 min to as long as one hour depending on the traffic flow and distance to the health units in Kampala. We found TB cases clustered in particular areas especially slum areas. Most of TB cases originate from slum areas. This observation may represent ongoing transmission in these areas. A study in high TB incidence urban setting in South Africa found 72 % of the cases were clustered within slum communities [31]. We found similar TB case notifications in the same slum areas over the two years of our report. This suggests the transmission cycle in those particular slum areas is uninterrupted.

### Strengths and limitations

This is the first report to document the burden of TB in Kampala from the routine programmatic data. We collected all the data reported in Kampala district during the review period for at least two years. At the minimum this would provide insight on TB epidemiology in children in Kampala district. We conducted this work before availability of Xpert® MTB/RIF in Kampala provides important comparative data in assessing the impact of Xpert® MTB/RIF wide use on TB detection in children.

We acknowledge some limitations to this work. We had two data points (2009 and 2010) which are not enough to show a trend in TB epidemiology constituting a selection bias. There is no comparative published childhood TB data for Kampala district to affirm our the observation as part of a national downward trend of TB incidence in Uganda [18]. We could not confirm the accuracy of the TB diagnoses. We report the bacteriologically confirmed cases based on only sputum smears which is an under estimate. Work done in South Africa found 22 % of the children with TB were smear negative but culture positive [27]. We used programmatic data that did not capture important aspects of paediatric TB epidemiology such as TB contact history, those on isoniazid preventive therapy and BCG vaccination status. The population projections we used depend on birth rates but for urban settings in Uganda population growth is mainly because in-migrations. Also population projects beyond 10 years become increasingly inaccurate. This is a reasonable explanation for the wide confidence intervals around our incidence estimates. We believe this is the best estimate within our limitations.

### Implication for practise, policy and research

This paper highlights the reality of under-reporting of childhood TB where documenting all childhood TB cases would improve the estimates. The sputum collection for TB detection in children was low and there is need to understand the underlying reasons. Assuming limited skills and knowledge of health workers as the explanation for low sputum collection in children may only be part of a larger problem. The finding that many children reside outside Kampala district but are diagnosed and treated in Kampala district requires further study. Contacts of smear positive cases were not captured in the unit TB registers representing missed opportunities for control of TB. The reality of large number of TB cases arising from the same slum areas means that targeted TB control interventions would break this cycle. In this study we noted the TB cases remained clustered in the same slum areas over the review period. The finding that 100 % of TB cases in the central division of Kampala resided in slum areas requires specific TB control interventions at household level. Using private health units in the slum areas to detect and treat TB has proved an effective intervention that is worthy strengthening. The Slum Partnerships to Actively Respond to Tuberculosis in Kampala (SPARK-TB) project showed impact of this approach by being able to identify an extra 1267 smear positive cases [32].

## Conclusions

There was a reduction in child TB incidence in Kampala district over the review period. The incidence was 56 per 100,000 in 2009 and 44 per 100,000 in 2010. The number of child TB cases was much higher at 7.5 % of all cases during the review period compared to the national average of 2.5 % in the world TB reports 2010, 2011. There was a high HIV co-infection rate and low anti-retroviral uptake over the review period. Pulmonary TB remains the commonest form of TB in children with children below five years bearing the biggest burden. For the review period, the TB cases clustered in particular Kampala district slum areas.
